# Middle ear malformations combined with cerebrospinal fluid otorrhea: A case report

**DOI:** 10.1097/MD.0000000000050719

**Published:** 2026-09-18

**Authors:** Ya Liu, Yaping Xu

**Affiliations:** a Department of Otolaryngology, The Second People’s Hospital of Hangzhou Yuhang District, Hangzhou, Zhejiang, China; b Department of Otolaryngology, The First Affiliated Hospital, Zhejiang University School of Medicine, Hangzhou, Zhejiang, China.

**Keywords:** cerebrospinal fluid otorrhea, conductive hearing loss, Middle ear malformations

## Abstract

**Rationale:**

Middle ear malformations (MEMs) encompass a diverse spectrum of congenital anomalies that significantly impair auditory function. They account for approximately 0.5% to 3% of conductive hearing loss cases, with etiologies involving both genetic and environmental factors. Clinically, MEMs complicated by cerebrospinal fluid (CSF) otorrhea are extremely rare.

**Patient concerns:**

A 17-year-old male presented with right-sided hearing loss of over 7 years’ duration. Otoscopic examination revealed a perforation in pars flaccid of the tympanic membrane. Pure-tone audiometry confirmed moderate conductive hearing loss in the right ear, while mastoid high-resolution computed tomography showed no obvious abnormalities.

**Diagnoses:**

Intraoperative observations revealed partial dehiscence of the vertical segment of the facial nerve canal, agenesis of the long process of the incus, and Hyrtl fissure with suspected CSF leakage from the bony defect.

**Interventions:**

The patient underwent tympanic cavity exploration surgery, during which the fissure was repaired, a partial ossicular replacement prosthesis was implanted, and the ossicular chain was reconstructed.

**Outcomes:**

At the 45-day postoperative follow-up, the tympanic membrane perforation was closed, conductive hearing loss improved, and the air-bone gap was markedly reduced.

**Lessons:**

To our knowledge, this represents the 1st reported case of MEMs complicated by presumed CSF otorrhea. The surgical intervention involving defect repair and artificial ossicle implantation significantly improved the patient’s quality of life.

## 1. Introduction:

Congenital middle ear malformations (MEMs) represent a heterogeneous group of embryonic developmental anomalies affecting the tympanic cavity, ossicular chain, and facial nerve. Clinically characterized by conductive hearing loss, these malformations occur with an incidence of approximately 1 in 10,000 individuals.^[1]^ Pathogenetically, they arise from aberrant development of the 1st and 2nd branchial arches and their associated germ layers, typically presenting unilaterally with a male predominance.^[2]^ Although MEMs are well documented in the literature, their coexistence with presumed cerebrospinal fluid (CSF) otorrhea (abnormal CSF leakage into the middle ear and external auditory canal) is extremely rare.

CSF otorrhea commonly results from temporal bone trauma, chronic inflammatory lesions, and congenital inner ear malformations, including Mondini dysplasia and incomplete partition deformity.^[3–5]^ Congenital CSF otorrhea derives from a deficiency of the bony barriers separating the intracranial cavity and middle ear compartment, such as a tegmen tympani defect, oval window, or round window structural defect, thereby disturbing cerebrospinal fluid circulation homeostasis.^[6]^ Notably, isolated MEMs without inner ear structural abnormality seldom induce CSF otorrhea. Subtle osseous fissures and latent structural weakness are easily neglected on conventional radiological examination, leading to missed diagnoses.

Herein, we retrospectively report a 17-year-old male patient presenting with long-term right conductive hearing loss. Intraoperative exploration confirmed typical middle ear malformations, including vertical nerve canal partial dehiscence and incus long process absence, accompanied by a bony fissure inferior to the round window and presumed CSF otorrhea. This rare entity belongs to isolated congenital middle ear malformation complicated with suspected CSF otorrhea without inner ear anomalies. Our case complements relevant clinical data and enriches literature evidence concerning complex otological congenital anomalies.

## 2. Case report

This clinical case study was approved by the local ethics committee (the institutional review board at the Second People’s Hospital of Hangzhou Yuhang District, Hangzhou City, China). Written informed consent was obtained from the patient for relevant clinical data and image publication.

**Figure 1.**
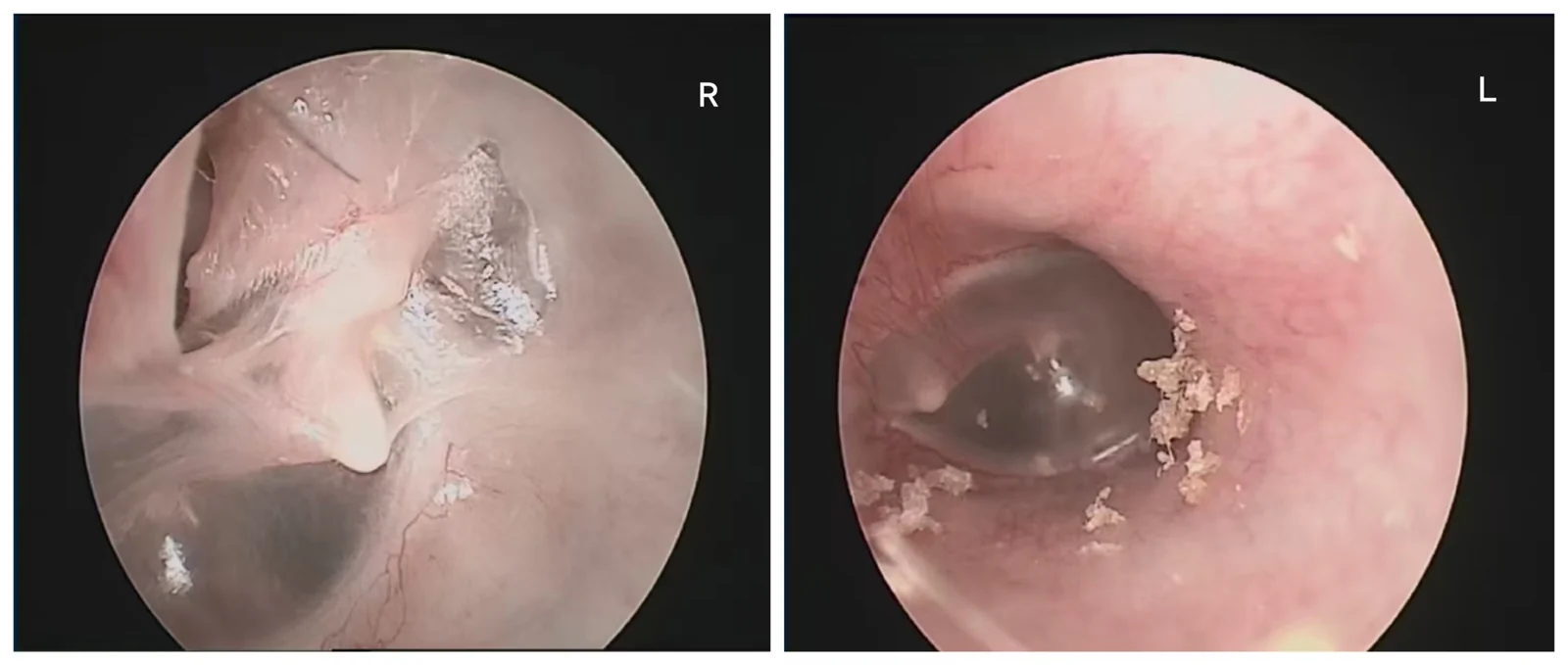
Otoscopic examination revealed a perforation in the flaccid part of the right tympanic membrane.

**Figure 2.**
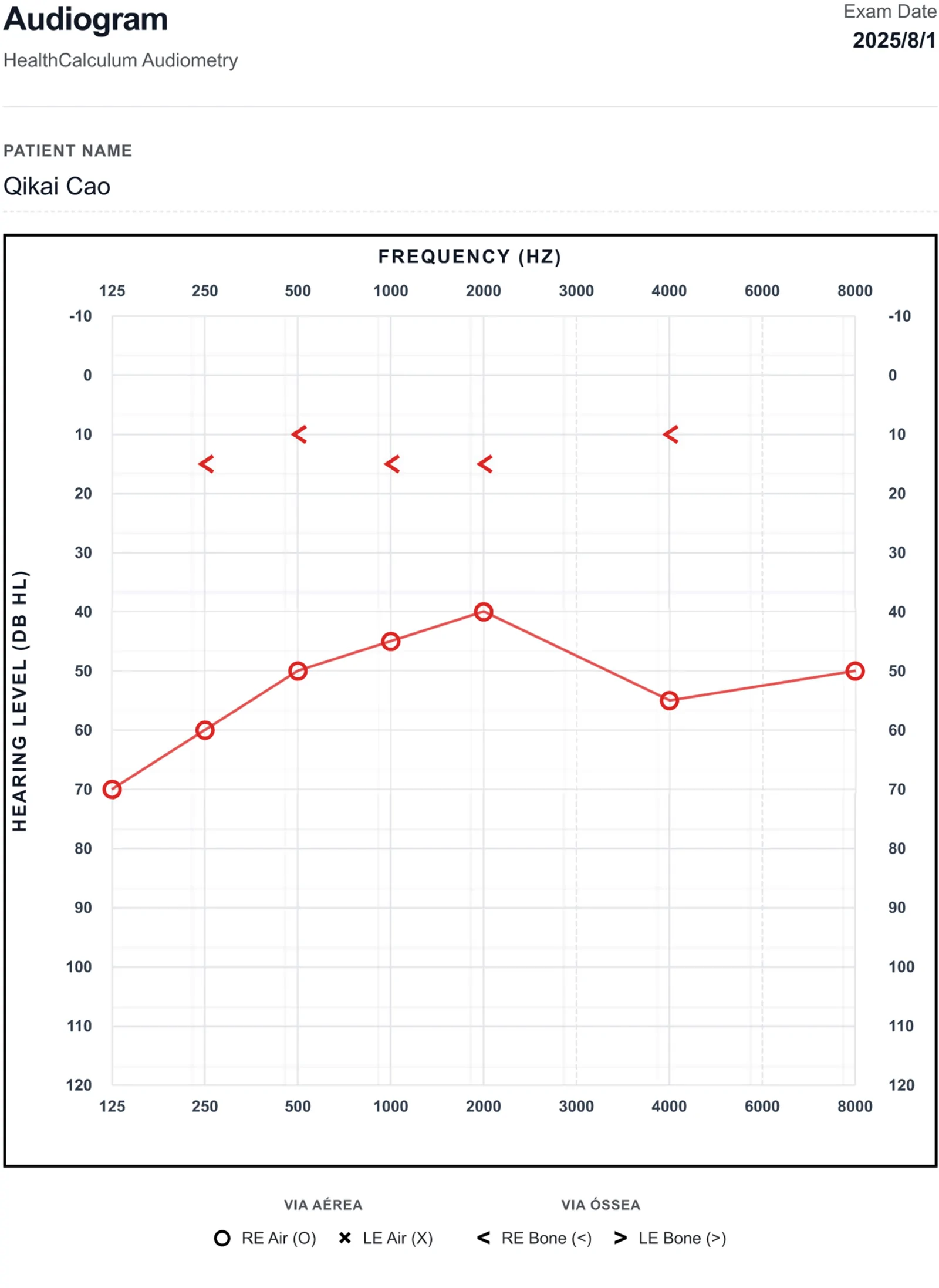
Pure-tone audiometry indicated moderate conductive hearing loss in the right ear and normal hearing in the left ear. LE = left ear, RE = right ear.

**Figure 3.**
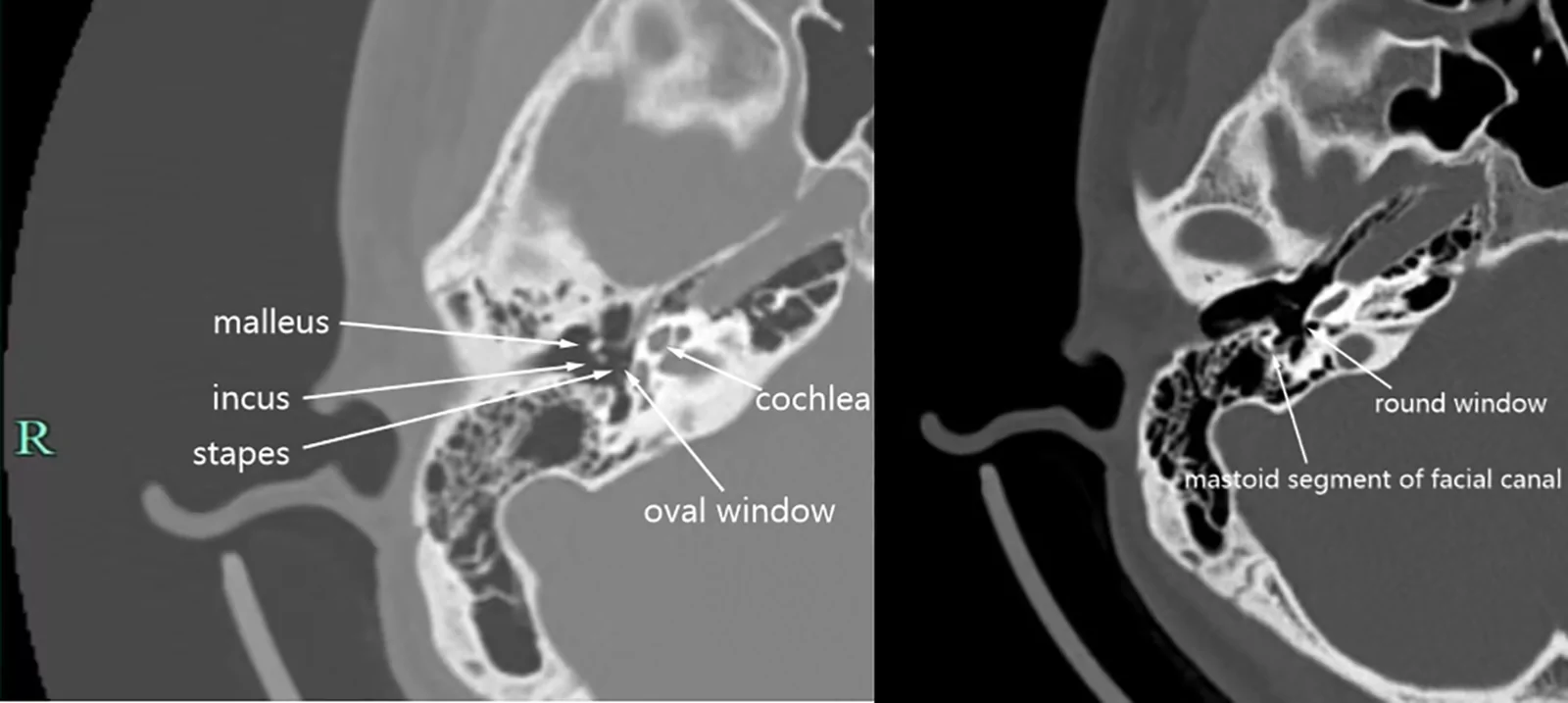
No obvious abnormalities were found on HRCT scan of the mastoid with 3-dimensional. HRCT = high-resolution computed tomography.

A 17-year-old male presented to our department with persistent right hearing impairment of >7 years’ duration. The patient initially suffered from right otorrhea accompanied by hearing loss, ipsilateral aural fullness, and intermittent tinnitus 7 years ago. No meningitis, otalgia, vertigo, nasal congestion, or rhinorrhea was complained. He previously received conservative treatment in a local hospital with a tentative diagnosis of chronic otitis media. Purulent ear discharge was gradually controlled, whereas hearing deterioration failed to be relieved. During the long-term follow-up period, recurrent otorrhea did not occur, and bilateral auditory condition remained stable without obvious progression or remission. No familial hereditary otological disease history and systemic comorbidity were recorded. External auricle appearance showed no congenital deformity. Otoscopic examination revealed right-sided tympanic membrane pars flaccida perforation, with no visible external auditory canal effusion or middle ear inflammatory exudate. The left tympanic membrane remained intact and normal (Fig. 1). Pure-tone audiometry confirmed moderate right conductive hearing loss (Fig. 2). Preoperative mastoid high-resolution computed tomography (HRCT) showed no definite abnormal bony lesion or suspicious leakage focus (Fig. 3). The scanning slice collimation was set to 0.625 mm.

**Figure 4.**
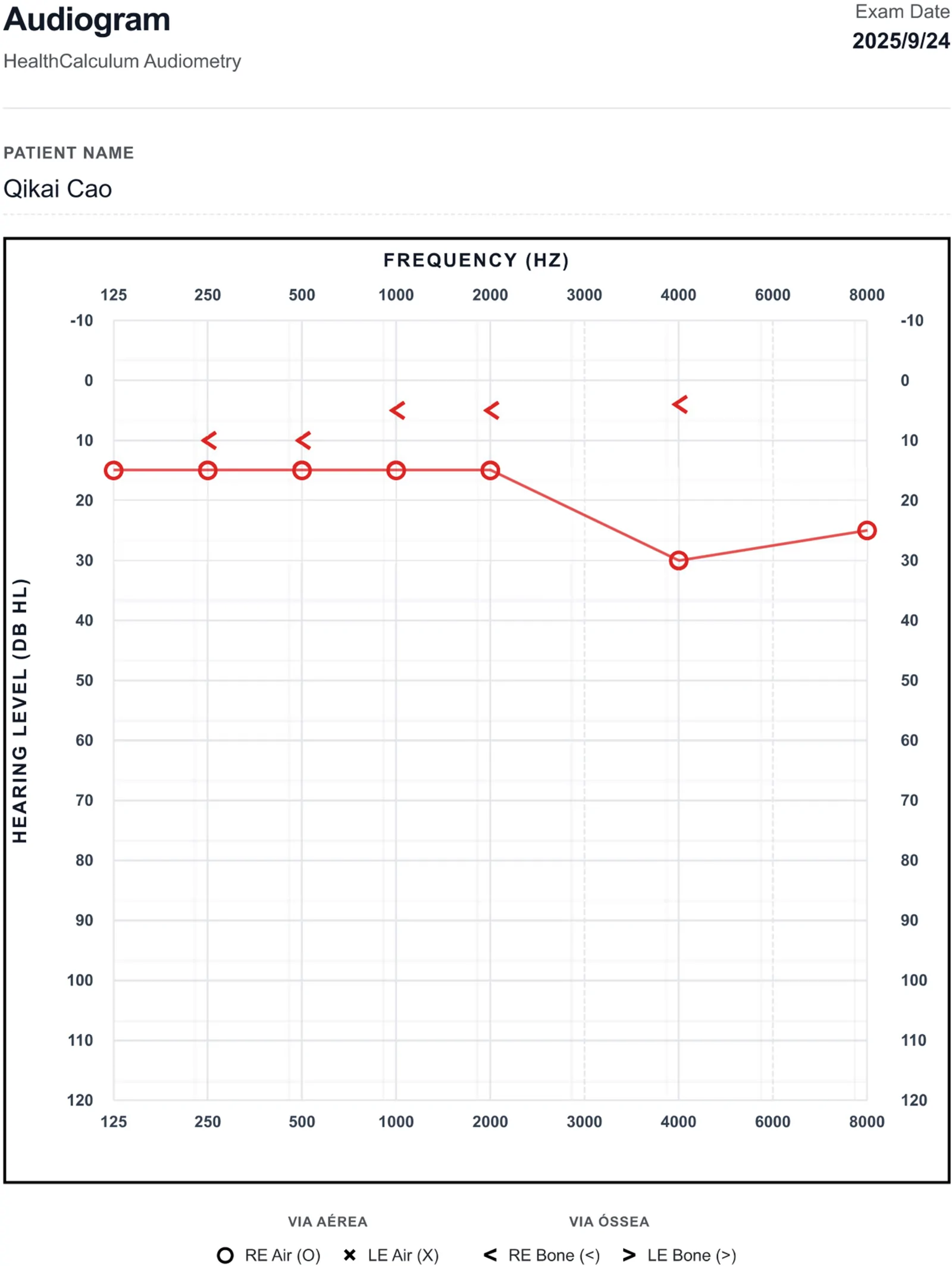
Pure-tone audiometry at the 45-day postoperative follow-up. LE = left ear, RE = right ear.

**Figure 5.**
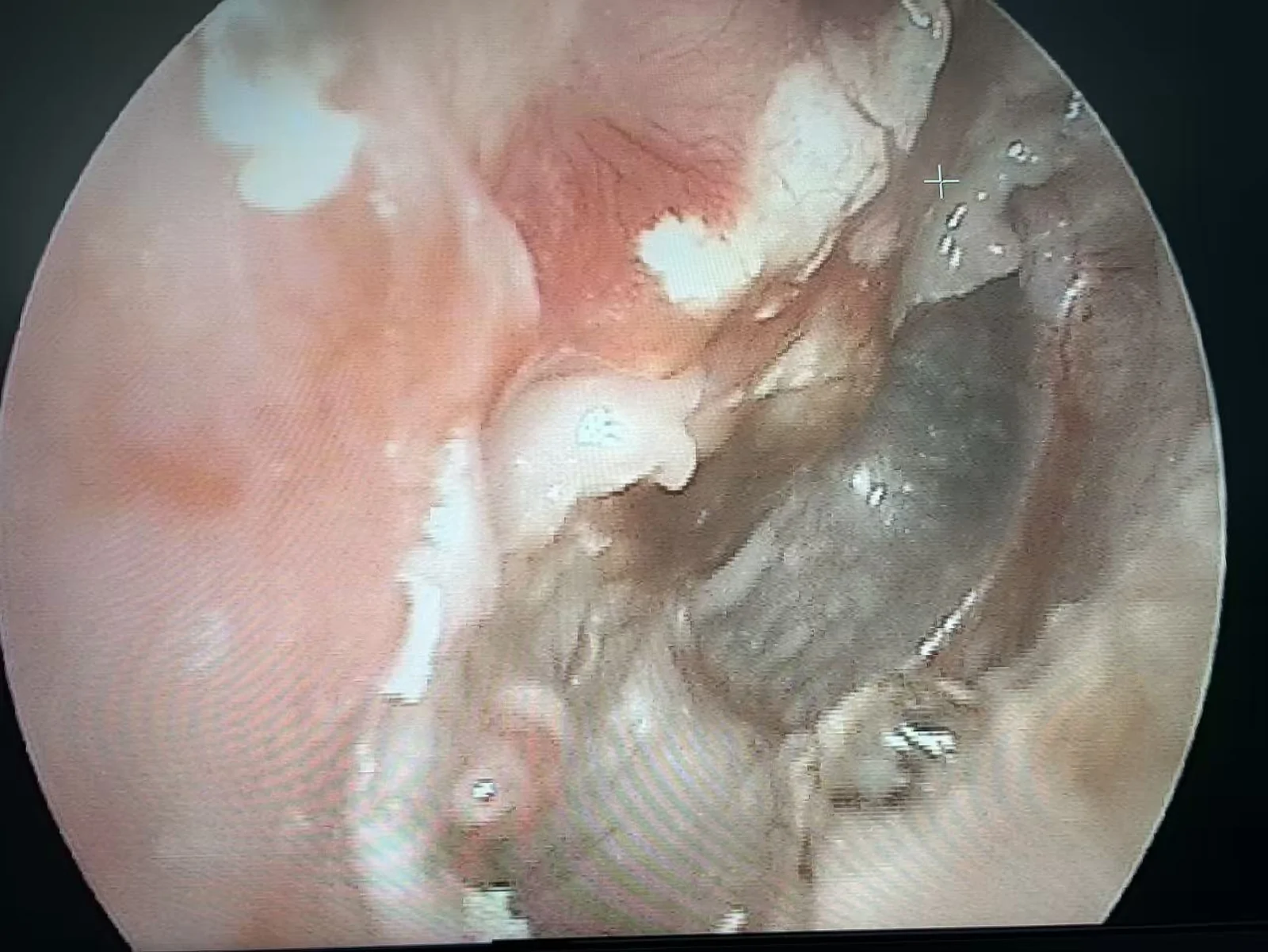
Otoscopic examination at the 45-day postoperative follow-up.

Surgery was performed under general anesthesia with microscopic assistance via a standard intra-auricular incision. Intraoperatively, dehiscence of the vertical segment of the facial nerve canal (approximately 0.5 cm in length) was observed, along with clear pale yellow fluid oozing (Video 1). Based on surgical outcomes and clinical assessment, CSF leakage is suspected. Upon exploration of the middle ear cavity, a bony fissure inferior to the round window was observed. Given its anatomical location matching Hyrtl fissure, we presume that this lesion resulted in CSF otorrhea. When examining the ossicular chain, absence of the long process of the incus was noted, while the stapes showed normal mobility. Temporalis fascia and fibrous tissue were harvested, trimmed, and implanted to occlude the bony fissure inferior to the round window. Absorbable gelatin sponge combined with biological fibrin glue was applied for secure sealing of the defect. Subsequently, partial ossicular replacement prostheses (PORP) was implanted to reconstruct the disrupted ossicular chain conduction pathway. Forty-five days after surgical intervention, regular outpatient follow-up was conducted. Postoperative audiometry showed nearly complete recovery of hearing across all frequencies in the right ear, amelioration of conductive hearing loss, and narrowing closure of the air-bone gap (Fig. 4). The reconstructed tympanic membrane remained intact and dry, with no recurrent CSF leakage or adverse otological symptoms (Fig. 5). The patient achieved substantial hearing improvement and an improved quality of life and was highly satisfied with the surgical results.

**Video 1.** 

## 3. Discussion

This report describes a 17-year-old adolescent presenting with unilateral conductive hearing loss and pars flaccida perforation. Preoperative HRCT demonstrated no definite bony destruction or pathological abnormalities of the middle ear and mastoid. Intraoperative exploration confirmed presumed CSF otorrhea originating from a bony fissure inferior to the round window, associated with agenesis of the long process of the incus and bony dehiscence of the facial nerve canal. The bony dehiscence inferior to the round window observed intraoperatively is anatomically confirmed as Hyrtl fissure, also known as tympanomeningeal fissure, an embryonal petrous bone cleft prone to incomplete ossification and serving as a congenital communication pathway between the subarachnoid space and middle ear cavity.^[7]^ Ossiculoplasty was performed during surgery, leading to remarkable postoperative hearing improvement with nearly complete closure of the air-bone gap. Given the patient’s clinical history featuring only a single transient episode of otorrhea in childhood, no recurrent suppurative otitis media, and absence of cholesteatoma or inflammatory granulation tissue intraoperatively, the case was definitively diagnosed as congenital middle ear malformation complicated with presumed CSF otorrhea, and acquired middle ear lesions secondary to inflammatory disease, trauma, or cholesteatomatous bony erosion were entirely excluded.

Chronic suppurative otitis media and middle ear cholesteatoma are the most common etiologies of acquired ossicular erosion, petrous bone dehiscence, and pars flaccida defect of the tympanic membrane. Sustained mucosal inflammation, granulation proliferation, and osteolytic activity of cholesteatomatous epithelium progressively erode the ossicular chain, tympanic cavity wall, round window region, and facial canal, resulting in bony resorption, anatomical disruption, and conductive hearing impairment.^[8,9]^ Typical clinical manifestations of such acquired lesions include recurrent otorrhea and progressive hearing loss; radiologically, HRCT demonstrates intratympanic soft-tissue opacification, moth-eaten bony destruction, and sclerotic mastoid air cells, with intraoperative findings consisting of inflammatory granulation tissue, cholesteatoma matrix, or chronic fibrotic scar.^[9]^ In contrast, the adolescent patient in this case had a prolonged disease course with only a single transient episode of otorrhea 7 years prior and no persistent middle ear infection. Preoperative high-resolution temporal bone CT, the gold standard for evaluating petrous bone pathology, revealed no inflammatory change or destructive bony lesion in the middle ear and mastoid. Intraoperative direct visualization showed smooth middle ear mucosa without cholesteatoma, granulation, adhesion, or osteosclerosis. In the absence of pathological evidence for inflammatory bony destruction, acquired structural damage secondary to chronic otitis media can be definitively excluded.

In the present case, facial nerve canal dehiscence coexists with agenesis of the long process of the incus and Hyrtl fissure as multiple concomitant middle ear structural anomalies, representing synchronous developmental defects. Such findings confirm that facial nerve canal dehiscence here is secondary to congenital middle ear malformation. In contrast, physiological dehiscence is typically an isolated incidental finding with an intact remaining ossicular chain and temporal bone architecture, which is inconsistent with the intraoperative findings of this case; thus, an isolated physiological variant can be excluded.^[10,11]^

MEMs represent a diverse group of congenital anomalies with significant implications for auditory function. Epidemiologically, MEMs account for 0.5% to 3% of all conductive hearing loss cases, with multifactorial pathogenesis involving genetic mutation and prenatal environmental exposure.^[12]^ Embryologically, the middle ear develops from the 1st and 2nd pharyngeal arches, structures derived from neural crest cells of the posterior midbrain and rhombencephalon. Specifically, the stapes originates from the 2nd pharyngeal arch, while the malleus and incus are derived primarily from the 1st pharyngeal arch. The inner ear derives from the ectoderm of the otic placode and develops. Disruptions in these developmental processes can lead to a spectrum of MEMs, ranging from minor abnormalities to severe malformations affecting several components of the middle ear.^[13,14]^ The embryonic origin and location of the inner ear are completely different from those of the middle ear; therefore, MEMs and Hyrtl fissure rarely occur simultaneously in clinical practice. However, in this case, both MEMs (partial exposure of the facial nerve and absence of the long process of the incus) and a congenital bony fissure inferior to the round window (Hyrtl fissure) leading to suspected CSF otorrhea causing presumed CSF otorrhea) were identified. Intraoperative drainage fluid was highly suspected to be CSF. The diagnosis of CSF otorrhea was preliminarily determined relying on intraoperative manifestations combined with comprehensive clinical judgment, without corresponding laboratory detection including beta-2 transferrin and beta-trace protein for further definitive verification.

Middle ear malformations may be associated with genetic and environmental factors. Studies have demonstrated that the FREM3 gene plays a crucial role in the formation of the external and middle ears during embryonic development, and its mutations are linked to human ear malformation syndromes – these mutations can lead to symptoms including lop ears, stenosis or atresia of the external auditory canal, and middle ear malformations. The protein encoded by the FREM3 gene is an extracellular matrix protein; its mutations typically follow an autosomal recessive inheritance pattern, which requires affected individuals to inherit the mutated gene from both parents to manifest the symptoms.^[15,16]^ Environmental factors encompass chemicals, pharmaceuticals, radiation, viruses, and more. Estriol (a follicular hormone), clomiphene (an ovulation-inducing drug), vitamin A, and thalidomide are all recognized as medications that induce congenital ear malformation.^[17,18]^

Preoperative imaging, particularly HRCT of the temporal bone, is critical for evaluating middle ear anatomy and potential CSF leak sites,^[19]^ but small bony defects or membranous covering of fissures may lead to false-negative results, necessitating intraoperative confirmation.^[20–22]^ The diagnosis of MEMs combined with CSF otorrhea poses significant challenges. Patients often present with long-standing conductive hearing loss due to middle ear structural anomalies, while CSF otorrhea may manifest as intermittent clear otorrhea – easily misdiagnosed as chronic otitis media – or remain asymptomatic until complications such as recurrent meningitis occur. In this case, preoperative mastoid HRCT showed no obvious abnormalities, with no obvious fluid leakage from the external auditory canal and no significant middle ear effusion. However, intraoperative confirmation revealed middle ear malformation and presumed CSF otorrhea. Three predominant mechanisms account for radiological underdiagnosis. First, microfistulous channels are obliterated by arachnoid membrane and fibrous connective tissues without aerated CSF filling, precluding CT-based delineation. Second, minute osseous defects of the ossicular chain and facial nerve canal remain masked by overlying mucosa or granulation tissue, with image degradation further induced by slice-thickness constraints and partial-volume artifacts. Third, fissures partitioned into multiloculated air cells are easily misclassified as physiological mastoid pneumatization. Asymptomatic remnant Hyrtl fissure typically manifests as isolated petrous apex air spaces, and clear fistular visualization on CT is only achievable under complete luminal patency with sustained CSF effusion. Consequently, occult fibro-osseous microfistulas are susceptible to radiological omission. Derived from aberrant embryonic otic capsule ossification, persistent Hyrtl fissure is frequently coupled with synchronous developmental dysplasias of the ossicular apparatus and facial nerve canal, compounding the risk of missed radiological detection.^[7,23]^ Based on previous clinical evidence,^[23,24]^ persistent Hyrtl fissure fissure with CSF fistula should be included in the differential diagnosis for patients afflicted with idiopathic recurrent serous otitis media, spontaneous clear otorrhea, or cryptogenic recurrent bacterial meningitis, even in the setting of normal preoperative temporal bone HRCT. Multimodality evaluation incorporating β2-transferrin quantification of otorrheal fluid and optional fluorescein lumbar cisternography is recommended to facilitate preoperative diagnosis. Intraoperative meticulous inspection of the anteroinferior round window niche, entire ossicular chain, and horizontal segment of the facial nerve canal is mandatory to preclude overlooked occult fistulous lesions.

Management of MEMs combined with CSF otorrhea requires a dual focus on repairing the CSF leak to prevent intracranial infection and reconstructing the middle ear conductive pathway to improve hearing. Surgical strategies for this condition include defect repair using autologous grafts, such as temporal fascia, abdominal fat, and ossicular chain reconstruction with prostheses.^[25,26]^ A recent pediatric study by Kortebein et al found that implantation of PORP can improve hearing function in patients with conductive hearing loss.^[26]^ In this case, the patient underwent PORP implantation, following which the hearing function of the right ear returned to near-normal levels. Given the paucity of reported cases, standardized treatment protocols remain underdeveloped, highlighting the value of documenting individual cases to guide clinical practice.

## 4. Conclusions

In summary, MEMs combined with presumed CSF otorrhea are rarely detected clinically. To our knowledge, this is the 1st reported case of MEMs complicated by cerebrospinal fluid otorrhea. For patients with conductive hearing loss suspected of MEMs, exploratory tympanotomy may reveal subtle pathological lesions even with negative preoperative HRCT findings. Surgical repair of the defect and implantation of an artificial ossicle are beneficial for improving the patient’s quality of life.

## Author contributions

**Conceptualization:** Yaping Xu.

**Resources:** Yaping Xu.

**Supervision:** Yaping Xu.

**Writing – original draft:** Ya Liu.

**Writing – review & editing:** Ya liu.


